# Supplementary material for: Late Post-Operative Occurrence of Dentin Hypersensitivity in Adult Patients Following Allogeneic Hematopoietic Stem Cell Transplantation—A Preliminary Report
Source: Int J Environ Res Public Health. 2021 Aug 19;18(16):8761. doi: 10.3390/ijerph18168761 (PMC8391522; doi:10.3390/ijerph18168761)
Supplement: Supplementary file 1 [file ijerph-18-08761-s001.zip › ijerph-1294485-supplementary.pdf]

DENTAL RECORD CARD

IDENTIFICATION NO. ___________________

AGE___________ SEX **F** **M**

PROFESSION: ____

1. GENERAL MEDICAL HISTORY.

Diagnosis (underlying pathology): ____

Date of HCT: ____

Date of dental examination:____

Graft type:

1. alloHCT ☐
2. autoHCT ☐

Type of conditioning therapy before HCT

1. Radiotherapy (TBI) ☐ 2. Chemotherapy ☐

Comorbidities:

GvHD disease (type):

Medications currently being taken:

2. DENTAL SELF-REPORT.

Pain conditions

Severity of pain (verbal scale) ☐ no pain ☐ mild ☐ moderate ☐ severe

VAS scale:

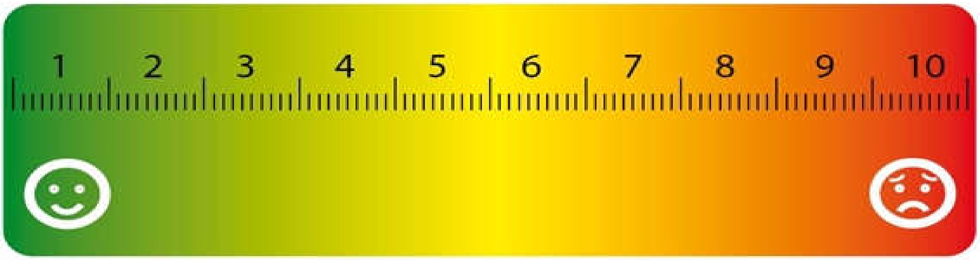

Which teeth are affected?____

What other parts of the oral cavity/throat are affected?

☐ lips ☐ cheeks ☐ palate ☐ gums ☐ tongue ☐ throat

What regions of the face are affected?

☐ lips ☐ cheeks ☐ nose ☐ forehead ☐ chin ☐ eye ☐ other

How long does the pain last?____

What causes the pain? ☐ cold ☐ heat ☐ sweet ☐ sour ☐ touch

What relieves the pain? ☐ cold ☐ heat ☐ touch ☐ other

Pain description:

☐ dull ☐ sharp ☐ throbbing ☐ spontaneous ☐ intermittent ☐ constant ☐ other

Problems with eating cold food	☐ no	☐ yes
The need for cutting fruits	☐ no	☐ yes
Careful when breathing	☐ no	☐ yes
Avoiding some teeth when eating	☐ no	☐ yes
Avoiding some teeth when brushing	☐ no	☐ yes

Type of pain

• Pain indicative of **dentin hypersensitivity**	☐ no	☐ yes
• Pain indicative of **pulpitis**	☐ no	☐ yes
• Pain indicative of **periodontal disease**	☐ no	☐ yes
• Pain indicative **neuropathic origin**	☐ no	☐ yes
• Other	☐ no	☐ yes

Dry mouth

A sensation of dry mouth 0 (none) ⟶ 10 (maximum severity)

How long has the patient been complaining of the condition?________________

Difficulty in swallowing 0 (none) ⟶ 10 (maximum severity)

How long has the patient been complaining of the condition?________________

Difficulty in speaking 0 (none) ⟶ 10 (maximum severity)

How long has the patient been complaining of the condition?________________

A frequent need to drink 0 (none) ⟶ 10 (maximum severity)

How long has the patient been complaining of the condition?________________

Dry mouth at night 0 (none) ⟶ 10 (maximum severity)

How long has the patient been complaining of the condition?________________

Dysgeusia 0 (none) ⟶ 10 (maximum severity)

How long has the patient been complaining of the condition?________________

Kind of dysgeusia: ☐ ageusia ☐ everything seems sweet ☐ sour ☐ bitter ☐ metallic

A burning mouth sensation 0 (none) ⟶ 10 (maximum severity)

How long has the patient been complaining of the condition?________________

A change in voice tone 0 (none) ⟶ 10 (maximum severity)

How long has the patient been complaining of the condition?________________

Halitosis 0 (none) ⟶ 10 (maximum severity)

How long has the patient been complaining of the condition?________________________________

Recurrent angular cheilitis 0 (none) ————————→ 10 (maximum severity)

How often does the patient complain of the condition?________________________________

Recurrent oral candidiasis 0 (none) ————————→ 10 (maximum severity)

How often does the patient complain of the condition?________________________________

Gum/periodontal disease

Bleeding when brushing teeth/flossing ☐ no ☐ yes

Retention of food in the interdental spaces ☐ no ☐ yes

Tooth mobility ☐ no ☐ yes

Gum recessions ☐ no ☐ yes

Oral hygiene and preventative care

Number of meals per day ________________________________

Most frequently consumed beverage________________________________

How many times does the patient brush the teeth a day?____________________

Kind of toothbrush: ☐ manual ☐ electric ☐ sonic

☐ ultra - soft ☐ soft ☐ medium ☐ hard

Type of toothpaste used ☐ with fluoride ☐ without fluoride ☐ high fluoride concentration

☐ smoker's ☐ teeth-whitening ☐ sensitivity

☐ herbal ☐ children's ☐ tartar-control

Other methods of oral care ☐ dental floss ☐ dental picks/sticks ☐ oral irrigator

☐ tongue scrapers ☐ other________________________________

Methods and measures for combatting dry mouth

☐ mouthwash________________________________

☐ oral gel________________________________

☐ moisturising spray________________________________

☐ artificial saliva________________________________

☐ chewing gum________________________________

☐ sweets________________________________

☐ other________________________________

Frequency of dental visits (a year)________________________________

Stimulants and habits

Coffee ☐ no ☐ yes

How often a day?______________________________

Strong tea ☐ no ☐ yes

How often a day? ______________________________

Tobacco ☐ no ☐ yes

How often a day? ______________________________

Alcohol ☐ no ☐ yes

How often? ☐ every day ☐ occasionally

Drugs ☐ no ☐ yes

How often? ☐ every day ☐ occasionally

Does the patient have any oral habits e.g. biting ice, biting nails, using teeth to hold objects? ☐ no ☐ yes

Does the patient grit their teeth during the day or cause them pain? ☐ no ☐ yes

Has the patient been diagnosed with bruxism? ☐ no ☐ yes

Prosthetic restorations/orthodontic treatment

Removable dentures:

☐ upper ☐ partial ☐ complete

☐ lower ☐ partial ☐ complete

☐ acrylic tissue-supported dentures

☐ dentures with cast metal frameworks

☐ dentures with cast metal frameworks and (semi)precision attachments

☐ other

Worn since____

Does the patient remove dentures at night ☐ no ☐ yes

How does the patient clean the dentures? ☐ water ☐ soap ☐ toothpaste ☐ special cleaners

Orthodontic/occlusal appliances:

☐ upper ☐ fixed ☐ removable

☐ lower ☐ fixed ☐ removable

☐ splints

☐ other___________________________________

Worn since____

3. CLINICAL ORAL EXAMINATION.

Extraoral examination

Limited mouth opening ☐ no ☐ yes (mm)____________________

Pain when opening ☐ no ☐ yes

Cracks on lips ☐ no ☐ yes

Angular stomatitis ☐ no ☐ yes

Other symptoms____

Intraoral examination

Oral mucosa changes:

☐ reticular pattern ☐ papular changes ☐ hypertrophic lesions

☐ erythematous spots ☐ white spots ecchymosis ☐ erosions and/or ulcers

☐ epithelial exfoliation ☐ mucocele ☐ other cysts

☐ scars

☐ other changes_________________________________

Location____

Oral mucosa hydration:

☐ **1º -** correct hydration

☐ **2º -** moderate dehydration

☐ **3º** - severe dehydration

Tongue:

☐ correct colour ☐ vivid ☐ red colour ☐ bluish

☐ dry ☐ moist ☐ swollen ☐ scalloped

☐ white coating ☐ black coating ☐ disappearance of filiform (filamentous) warts

☐ erosions/ulcers ☐ fissures ☐ pseudomembranous slough

☐ limited mobility

Salivary ducts: ☐ swelling ☐ erythrema ☐ pain

Periodontal status: (www.periodontalchart-online.com, Department of Periodontology, University of Bern, Switzerland)

Dentin hypersensitivity chart (DH):

Cold air																
Dental probe																
Tooth	18	17	16	15	14	13	12	11	21	22	23	24	25	26	27	28
Tooth	48	47	46	45	44	43	42	41	31	32	33	34	35	36	37	38
Dental probe																
Cold air																

0-no pain
1-discomfort
2-pain occurs with stimulus but resolves when it is removed
3-pain lasts after removal of stimulus

Dental chart:

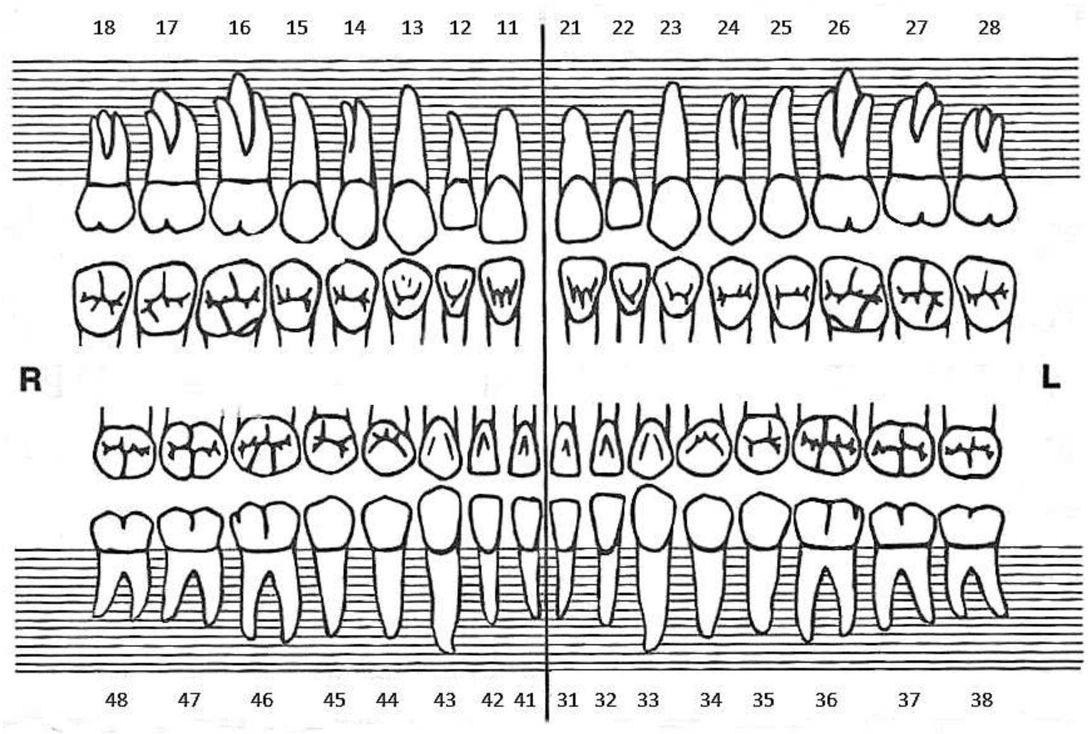

1 – initial caries
2 –caries
3 – non-carious lesion
4 – filling
5 – tooth extracted due to caries
6 – tooth extracted for other reasons
7 – fissure sealant
9 – partially erupted tooth
9 – trauma

Saliva

Appearance of saliva:

☐ correct appearance and quantity

☐ correct appearance but low quantity

☐ frothy and low quantity; cloudy; stretchy

Saliva secretion:

Resting saliva [ml]	
Stimulated saliva [ml]	
